# Signal preprocessing for foreign body detection using terahertz real-time non-destructive imaging system

**DOI:** 10.1371/journal.pone.0319978

**Published:** 2025-06-27

**Authors:** Ha-Neul Lee, Yuna Park, Jong-Tae Park, Jong-Ryul Yang

**Affiliations:** 1 Department of Electrical and Electronics Engineering, Konkuk University, Gwangjin-gu, Seoul, Republic of Korea; 2 Millisight Technologies Co., Ltd., Gwangjin-gu, Seoul, Republic of Korea; King Fahd University of Petroleum & Minerals, SAUDI ARABIA

## Abstract

A signal preprocessing method is proposed for foreign body detection using a terahertz real-time, nondestructive imaging system. To ensure the precise detection of foreign bodies in objects on a moving conveyor belt, it is crucial to calibrate and normalize the nonuniformities arising from the conveyor belt geometry, transmitter beam profiles, and receiver responsivities in terahertz transmission-based nondestructive imaging systems. The proposed method normalizes the nonuniformities of the image using data between objects on the conveyor belt without the need for characteristic compensation processes. Moreover, it enables continuous calibration of background characteristics, which may fluctuate during real-time image acquisition. Furthermore, this method performs image reconstruction to match the shape of an object by identifying the position of the object on the conveyor belt and acquiring only the positional data. This approach efficiently extracts the region of interest, facilitating time-efficient and precise discrimination of foreign bodies within objects in AI-based image processing. Experimental results obtained from three samples using a 0.2-THz imaging system demonstrate that the proposed method effectively isolates the object shape, improves the signal-to-noise ratio of the processed images by an average of 6.0 dB, and reduces the Fréchet inception distance by an average of 16.7%.

## Introduction

Transmission imaging systems that employ sub-terahertz signals above 200 GHz (hereafter referred to as terahertz (THz) imaging systems) are effective in detecting non-metallic materials, plastics, carbides, and other substances that are not discernible by conventional vision, X-rays, or metal detectors [1–4]. A distinguishing feature of THz imaging systems is their capability to generate transmission images based on the disparity in dielectric properties among materials and the penetration characteristics of electromagnetic (EM) waves. This capability sets them apart from imaging systems that utilize cameras, which are limited to surface inspection [1–4]. Furthermore, THz systems exhibit a superior capacity to facilitate precise imaging of nonmetals when compared to metal detectors [5]. The low ionization energy of the terahertz signal minimizes its impact on biological tissue, food and organic matter compared to X-rays. THz imaging systems are promising solutions for real-time foreign object detection in food production environments [6,7].

A THz imaging system employing continuous-wave signals quantifies the signal intensity detected at a detector array situated opposite the object as the transmitted signals traverse the object [8]. The reconstruction of objects passing through a conveyor belt into a two-dimensional image is based on the signal magnitudes acquired by detectors arranged perpendicular to the direction of travel [9]. Reconstructed THz images provide information regarding the internal and external geometries of nonuniform objects, as well as the differences in the dielectric properties of the materials composing the object. Such data may be employed to identify the presence of foreign objects or defects and to characterize nondestructive testing procedures [10]. The ability of THz imaging systems to detect foreign objects on conveyor belts in real-time is a significant advantage. This capability enables the removal of extraneous materials from the production process or the identification of defective products, thereby ensuring product quality control, safety, and reliability with minimal disruption to the throughput of the production line [11].

THz imaging systems encounter several limitations that arise primarily from the inherent characteristics of electromagnetic (EM) waves at the operating frequency, including scattering, diffraction, and other related phenomena. Additionally, variations in the geometry and dielectric properties of target objects further complicate the imaging process. These factors result in image quality that is lower in precision and resolution than those achievable with optical cameras. To address these challenges, many researchers have investigated methods to enhance transmitter output, including the use of array structures or vacuum devices [12–15]. Additionally, they explored techniques to improve the hardware performance of the system, such as incorporating large-scale detectors and advancing detector sensitivity [16–19]. Despite these improvements in transmitter and detector functionality, the intrinsic nature of EM wave propagation, which is constrained by the heterogeneous and non-uniform characteristics of objects, renders achieving a definitive solution challenging. Configurations that increase the operating frequency to alleviate the resolution limitations imposed by EM wave properties face two significant challenges. First, frequency-dependent differences in water absorption affect the physical properties of a system [20,21]. Second, performance constraints exist on the devices and integrated circuits that can implement these configurations [8,22]. Artificial intelligence (AI) has been applied to image analysis to examine the data from THz imaging systems. However, these technologies encounter difficulties when examining and analyzing comprehensive, real-time images of objects in motion along production lines [23,24]. This necessitates the implementation of preprocessing techniques capable of effectively extracting regions of interest (ROI) and utilizing AI within those regions.

In this study, a signal preprocessing technique is proposed to rapidly obtain ROI in unstructured, inhomogeneous objects traveling on a conveyor belt. The proposed technique employs a selective extraction process that isolates the region in which the object is situated within the acquired image. The optimization and preprocessing of images can significantly reduce the computational and memory demands associated with image processing. This is accomplished by reducing the ROI to a more compact dataset than the raw images acquired from the entire detector array. In addition, the technique calibrates distortion effects caused by the presence of objects on the conveyor belt and the belt itself. The proposed technique effectively identifies atypical, non-uniform objects within a circular sample tray, enhances the signal-to-noise ratio (SNR) of the acquired images, and reduces the Fréchet inception distance (FID) when applied to a THz imaging system utilizing commercially available 0.2-THz transmitting and receiving modules.

## Materials and methods

**Fig 1:**
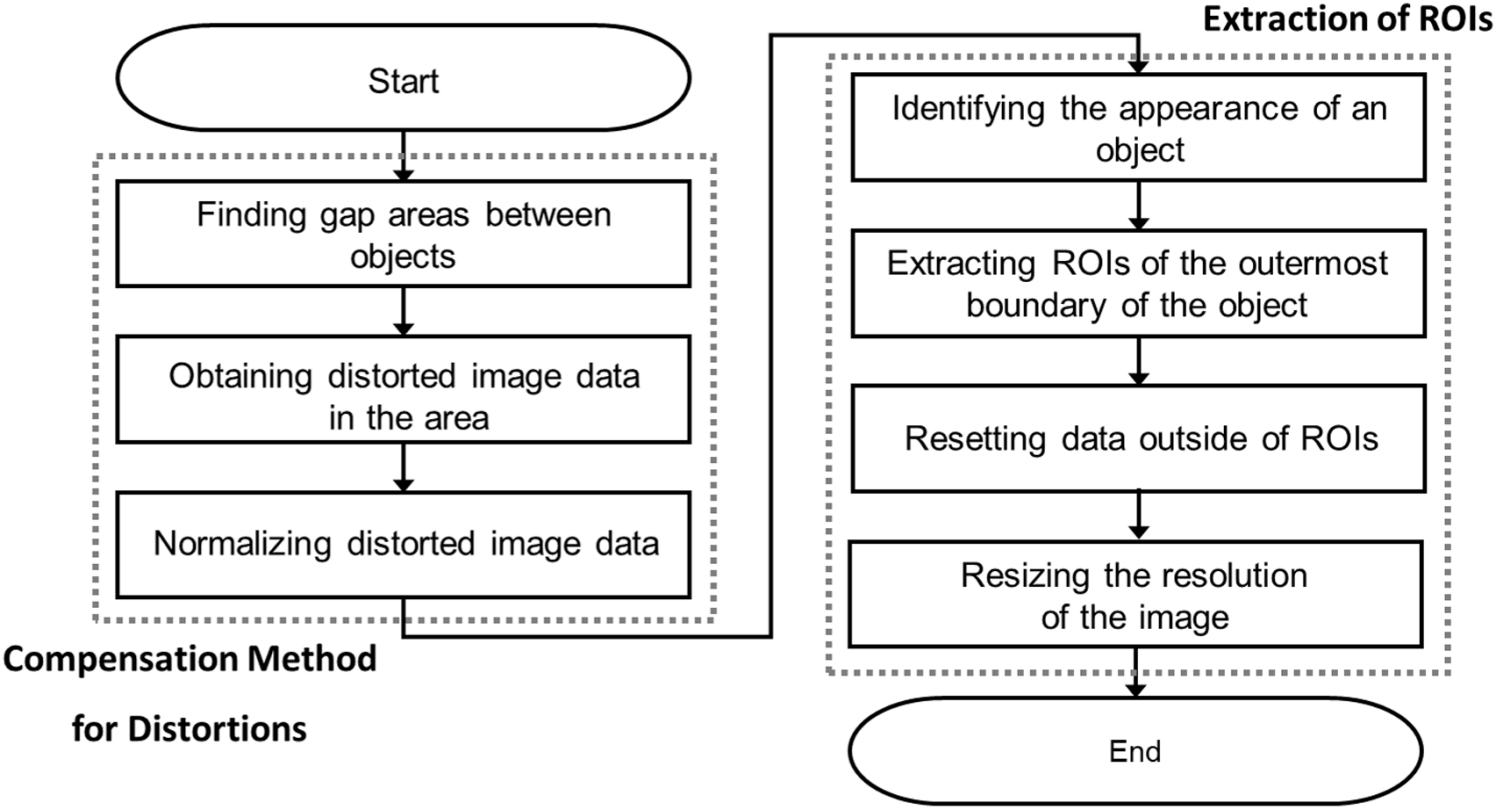
Proposed signal preprocessing procedure of the THz see-thru imaging.

The proposed preprocessing technique consists of two steps: compensation for the distortions caused by the conveyor belt and non-uniform transmitter beam profile, and ROI extraction, which selectively locates and reconstructs only the region where the object is located in the compensated image. Fig 1 shows the flowchart of the steps in the overall signal preprocessing algorithm.

### Compensation method for distortions in THz images

THz transmission imaging of an object placed on a conveyor belt inherently includes information regarding the changes in EM wave characteristics induced by the conveyor belt as THz waves propagate through it. A common method for compensating for the characteristic variations introduced by conveyor belts involves an independent calibration process, typically conducted using data acquired in environments without objects [17]. However, this approach has several limitations. First, the weight of the objects placed on the conveyor belt can induce deformation, altering the shape of the belt. In addition, it is often impractical to assume that the objects on the belt are uniform in shape and material properties, which introduces further variability. Consequently, conventional compensation methods struggle to effectively account for these dynamic changes. Second, the joint sections of the conveyor belt, which may vary in thickness and material composition compared with the rest of the belt, present another significant challenge [25]. Assuming the belt to be a uniform medium, as in conventional approaches, can lead to image distortions and inaccuracies owing to the inherent nonuniformity of the belt material and structure.

**Fig 2:**
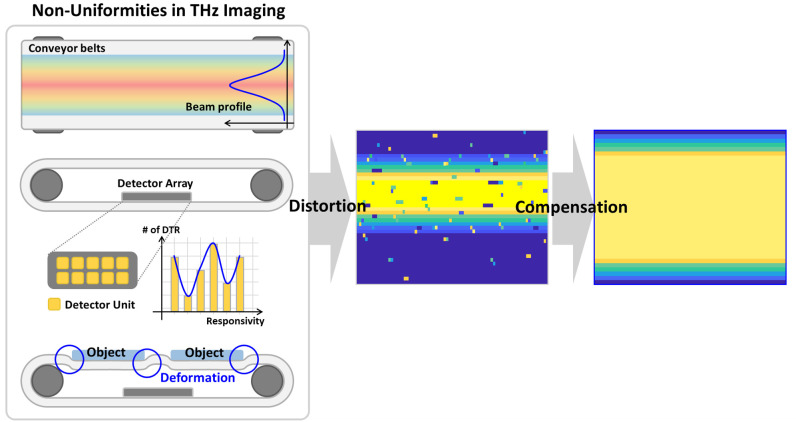
Image normalization techniques to compensate for non-uniformities in the measurement environment and transmitter/detector array characteristics.

As depicted in Fig 2, the proposed method for compensating the profile distortions caused by the conveyor belt, non-uniform beam characteristics, and detector responsivity relies on image normalization. This method utilizes data acquired from the gaps between objects in the THz imaging system to correct conveyor-belt distortions. When the separation between the objects exceeds the horizontal resolution of the imaging system, the information from these regions reflects the deformation characteristics of the belt induced by the weight of the object. These data serve as critical references for improving the accuracy of the compensation process. To further refine the compensation, characteristic data are collected from both the leading and trailing gaps of the objects on the conveyor belt, and the results are averaged. However, nonuniformities may still arise in the received data owing to factors such as localized focusing or spreading of the transmitted beam, unequal sensitivity within the detector array, or limitations in the dynamic range of the detectors. These issues can lead to inconsistencies in the signals obtained from gaps between objects [1 8]. Rather than individually addressing each source of distortion, the proposed method focuses on normalizing the final acquired images to ensure overall uniformity. This is achieved by applying weighted gain factors to correct cumulative distortions, regardless of their specific origins. Although this method requires a detector array with a high dynamic range to achieve optimal results, it demonstrates significant improvements in image quality under appropriate hardware conditions.

### Extraction of regions of interest

**Fig 3:**
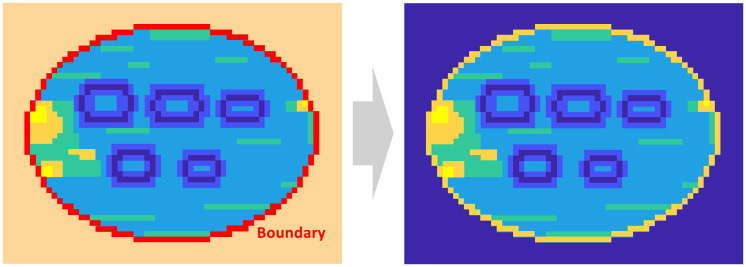
Conceptual illustration of the region-of-interest (ROI) extraction process.

The extraction of ROIs for objects passing through the field of view of a THz imaging system on a conveyor belt is based on the principle that THz signals incident on the detector array exhibit the most significant variations at the outermost boundary of the object contour. The transmitted THz signals exhibit the most pronounced scattering and diffraction characteristics at the outermost boundaries of the shape of the object, with notable canceling and compensation for the interference effects occurring around these boundaries. Fig 3 illustrates the ROI extraction process.

The proposed method utilizes these characteristics to identify regions within the obtained image where the received data between neighboring pixels exceeds a specific threshold. This threshold is typically determined using the mean dynamic range. These regions are subsequently evaluated for continuity and connectivity and are classified as shapes if they meet the specified criteria. Furthermore, this method extracts the outermost shapes from the overall area relative to the image center into an ROI, thereby mitigating the impact of noise introduced by individual pixels. When an object is assumed to have a generalized shape, such as a circle, an ellipse, or a rectangle, its shape can be extracted by approximating the continuously connected boundary to the corresponding generalized shape. The region within the extracted boundary is designated as the ROI, and the data outside this region is set to either the minimum or maximum value. This process eliminates dynamic regions or areas that negatively impact the SNR of the image and accelerates the computational processing of the inner regions in subsequent computations.

**Fig 4:**
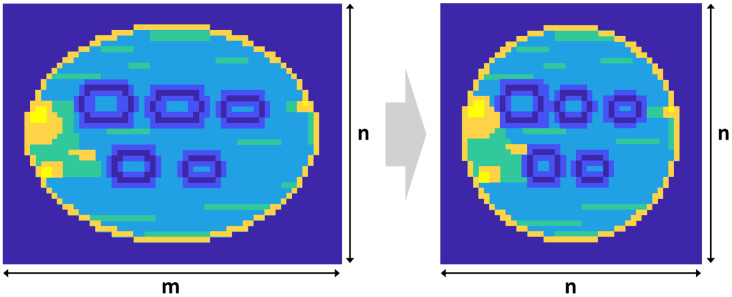
Image resizing for equalizing horizontal and vertical resolution scaling in THz images with different horizontal and vertical resolutions.

Following the establishment of the ROI, extraneous data unrelated to the appearance of the object are filtered out, and the acquired image is reconstructed to conform to the frame size required for subsequent image processing. In the reconstructed image, the x-direction corresponds to the moving direction of the object along the conveyor belt, whereas the y-direction represents the width of the belt. The resolution of a single-frame from the raw signal is determined by two factors: the resolution in the x-direction, which depends on the movement speed of the conveyor belt and the data acquisition rate of the detector array; and the resolution in the y-direction, which is dictated by the number of detectors in the array of the THz imaging system. Reconfiguring the data into a square matrix is advantageous because it facilitates matrix operations commonly used in AI-based image processing [26]. As shown in Fig 4, in the proposed technique, the horizontal and vertical pixel dimensions of the ROI are adjusted to achieve an n × n resolution, where the horizontal resolution is mainly determined by the conveyor speed. The values of the horizontally expanded pixels resulting from the enhanced resolution are interpolated by averaging the values of the stretched pixels.

The possibility of arbitrary resolution adjustment arises from the capability of the obtained THz image to represent the geometry of the object relative to the field of view of the detector array. However, it is not feasible to obtain the physical size directly from the detection signal acquired by the THz imaging system alone, because the physical size of the sample and the distance between the transmitting and receiving ends vary in real-world environments. To obtain the physical sizes, a standard sample must be measured, and the physical size must be compensated accordingly. Thus, an arbitrary resolution adjustment process was integrated to expedite the image processing technique. When the physical size is required for measurement, it is possible to generate an image depicting the absolute object shape by adjusting the resolution of the original signal based on the relative ratio to the standard sample for size compensation, followed by the application of the proposed method.

### Configuration of the THz imaging system

**Fig 5:**
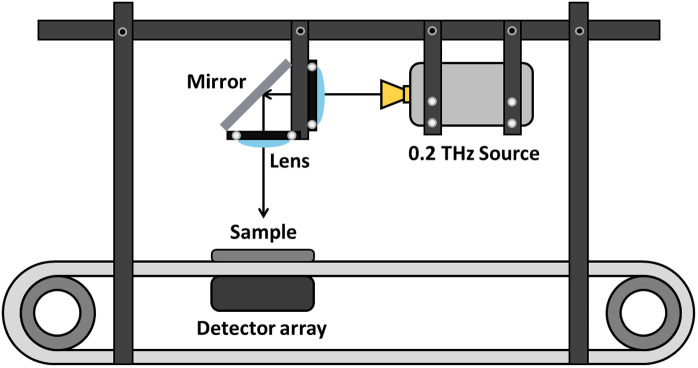
Configuration of the THz imaging system used to validate the proposed preprocessing method.

The THz imaging system, shown in Fig 5, comprises a 0.2-THz single-unit signal source and a 1 × 256 detector array, both manufactured by Terasense. The system also includes an optical assembly, a conveyor belt moving at a speed of 0.24 m/s, and a data acquisition unit, all of which are used to validate the proposed preprocessing method. A 200-mW signal generated by the 0.2-THz source passes through the optical system and impinges perpendicularly on a conveyor belt with a width of 310 mm. The detector array, with an effective irradiation width of 200 mm, is positioned beneath the conveyor belt, and the incident signals are converted into output voltages, which are then transmitted to a data acquisition unit. The acquired data are processed on a PC to generate an image that focuses on the region where a single sample is present. However, the captured image may experience horizontal distortion from its original shape because of the motion of the conveyor belt and the sampling and storage rates of the data acquisition unit.

**Fig 6:**
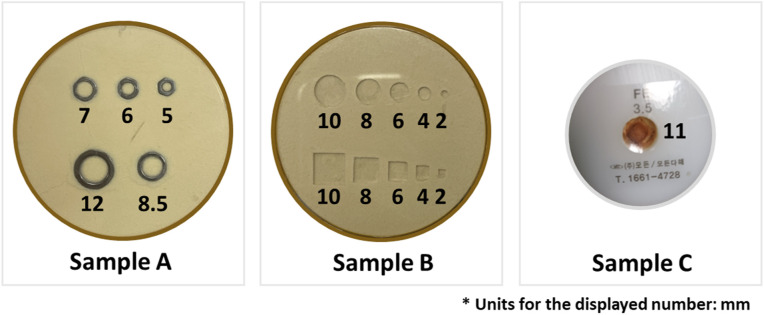
Photographs of the samples under inspection.

The samples under inspection consist of circular plastic trays containing grain powder and various foreign objects, as illustrated in Fig 6. Samples A and B are circular trays of identical dimensions, each measuring 65 mm in diameter, containing grain powder mixed with various randomly distributed metal and plastic objects of different sizes and shapes. Sample A includes five hexagonal nuts with varying internal diameters and thicknesses of 1 mm. Sample B contains five foreign plastic bodies of different diameters and shapes, including round and square forms with distinct length-to-side ratios. In contrast, Sample C is a circular Teflon shell with a diameter of 45 mm, and contains a centrally located sintered metal powder with a diameter of 11 mm. The dimensions of each foreign object, including its diameter and side length, are shown in Fig 6.

### Implementation of the proposed signal preprocessing

**Fig 7:**
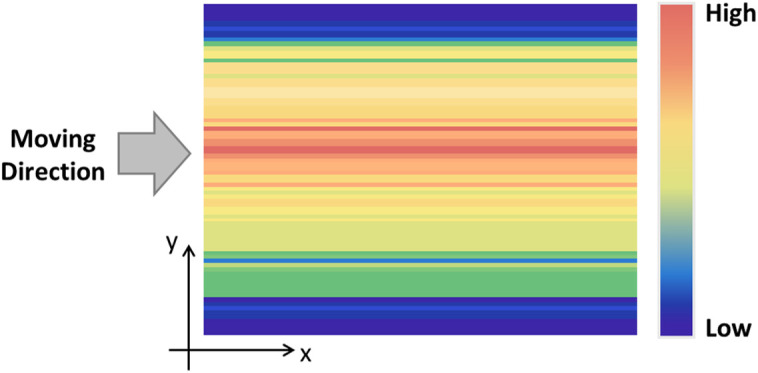
Beam profiles of the conveyor belt without samples.

Internal ROI extraction, based on the assumption of a circular sample exterior, utilizes data from the acquired circular regions to compensate for overall distortions caused by nonuniformities, such as deformations from the conveyor belt and variations in the beam profile. The output from the THz source passes through the optical system and is transformed from a point source into a line beam source. However, owing to optical system limitations, the beam profile across the conveyor belt, as illustrated in Fig 7, exhibits the highest intensity at the center of the belt, with a rapid decrease in intensity toward the outer perimeter. Conventional THz imaging systems employ a data compensation technique in which a beam profile measured without the sample, as shown in Fig 7, is used to correct inhomogeneities in the beam profile. However, this method does not address the image distortion resulting from conveyor belt deformation caused by the weight of the sample. To overcome this limitation, the proposed method captures beam profiles from the spaces between samples on the conveyor belt and averages them to compensate for the images of adjacent samples. In the distortion-compensated images, the ROI is extracted by approximating the boundary of the circular tray as an ellipse and defining the boundary as the point at which the outputs from the detector array exhibit a sharp variation.

The assumption of an elliptical boundary stems from the distortion of the circular tray-shape owing to the resolution of the acquired image. In the images from which the ROIs were extracted, all outer data points of the ROIs were set to zero to reduce the matrix computation time. The ROI was reconstructed with the same resolution as the original signal by identifying the boundaries where zeros appeared more than four times consecutively in both rows and columns. The resolution was 256 × 256 pixels, corresponding to the number of vertically arranged detectors. The ROI-reconstructed image was then normalized and converted to the data format of uint16, as required for subsequent signal processing.

### Measurement results and discussion

**Fig 8:**
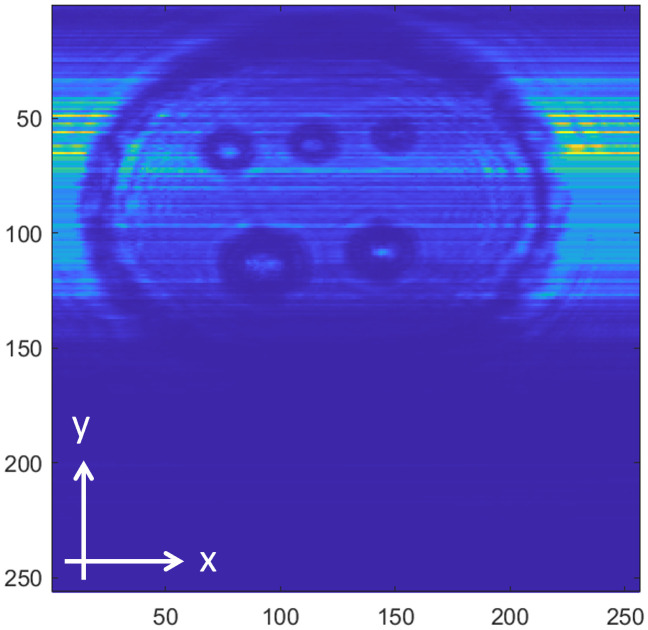
THz raw image of Sample A.

**Fig 9:**
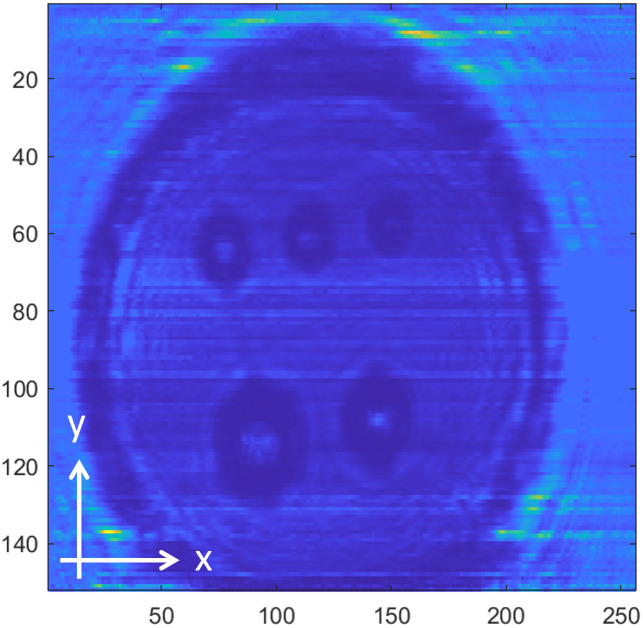
Processed image of Sample A using the compensation method for distortion and extraction of ROI.

**Fig 10:**
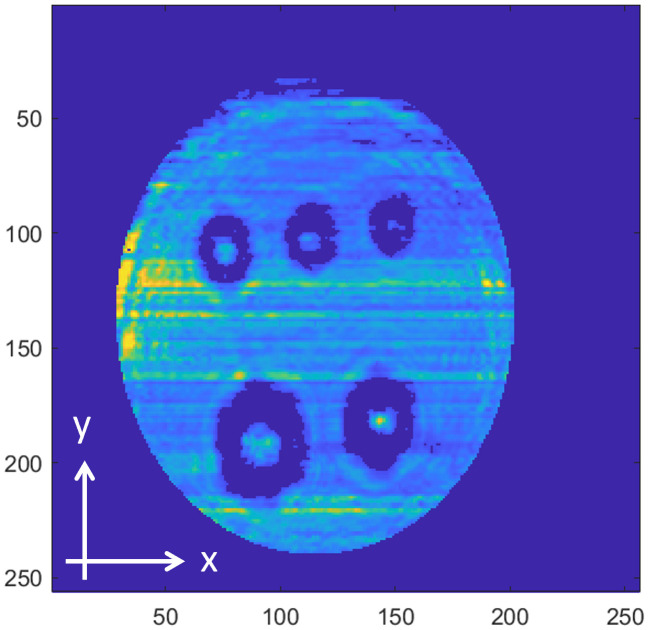
Processed image of Sample A using the overall proposed method.

**Fig 11:**
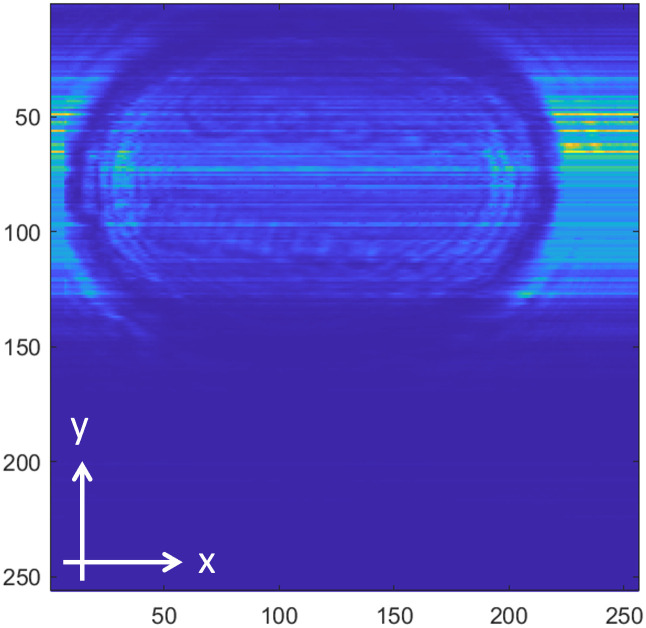
THz raw image of Sample B.

**Fig 12:**
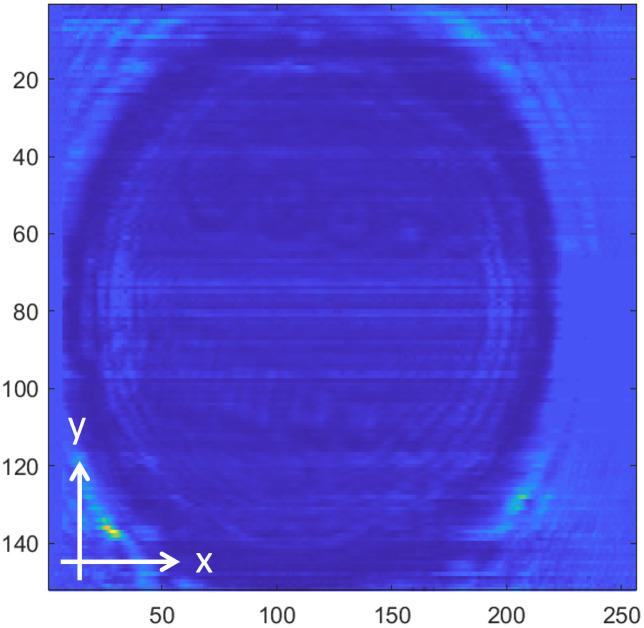
Processed image of Sample B using the compensation method for distortion and extraction of ROI.

**Fig 13:**
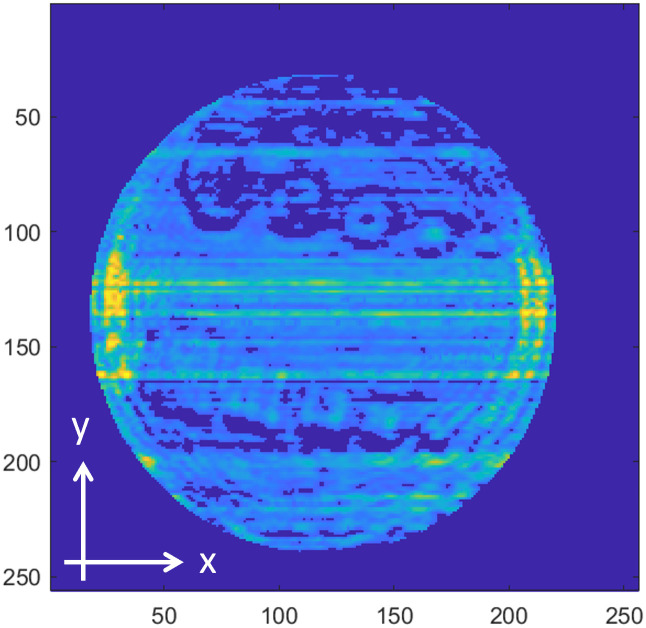
Processed image of Sample B using the overall proposed method.

**Fig 14:**
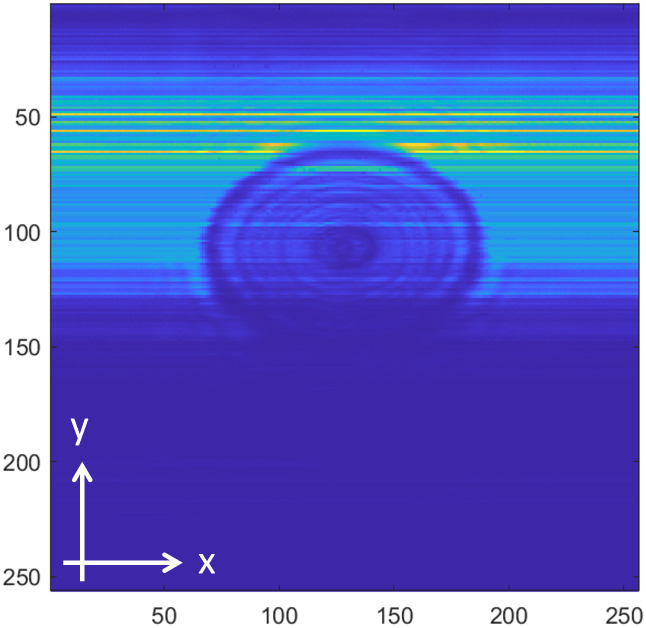
THz raw image of Sample C.

**Fig 15:**
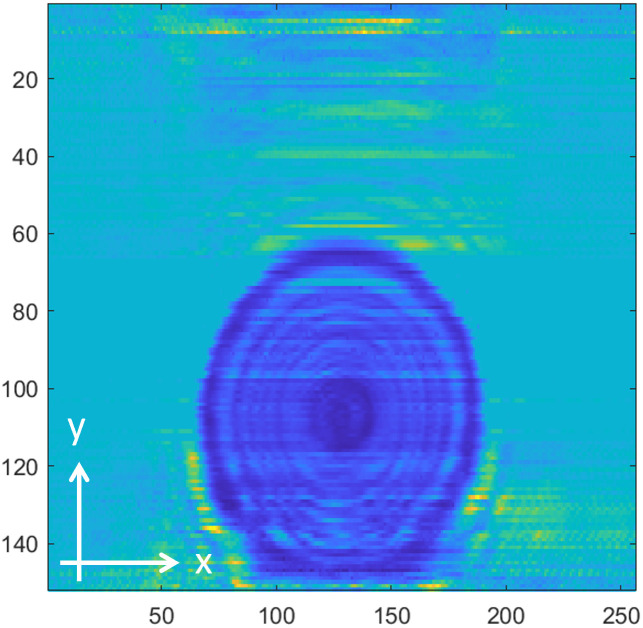
Processed image of Sample C using the compensation method for distortion and extraction of ROI.

**Fig 16:**
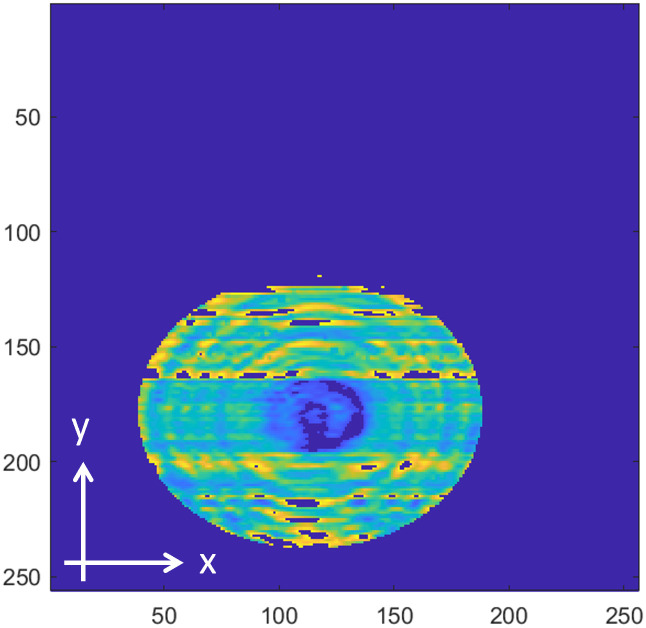
Processed image of Sample C using the overall proposed method.

Figs 8−16 shows the processed signals obtained using the proposed method for the three samples, along with the raw images captured by the THz imaging system. When the transmitter output is obstructed by an object on the conveyor belt, the geometry of the object becomes visible in the regions where the detector output voltage is low. Compared to the raw images reconstructed directly from the detector array outputs, as depicted in Figs 8, 11, and 14, the processed images generated using the proposed method, presented in Figs 10, 13, and 16, demonstrate successful extraction of the interior structure, regardless of the tray size. The raw images reveal a non-uniform beam profile perpendicular to the movement direction of the conveyor belt. The detector output shows a pronounced peak around the maximum value, with a gradual decline in intensity toward the edge of the conveyor belt. The distortion of the beam can be modeled and compensated using a mathematical beam profile, such as a Gaussian function. However, modeling the exact beam distribution is challenging because it depends on various factors, including the distances between the transmitter, optical components, and detector array, as well as the position of the conveyor belt. In the proposed method, a linear normalization technique is employed to calculate the gain for each pixel, thereby normalizing the non-uniform images to a uniform state within the beam coverage area where no sample is present. These normalized gains are then applied by multiplying them with the corresponding pixel outputs. As illustrated in Figs 9, 12, and 15, the proposed method achieves distortion compensation, including beam profiles, and ROI extraction. Figs 8−16 presents the normalization results for 256 vertically arranged pixels, demonstrating the effectiveness of the linear normalization technique in compensating for the beam profile distortion.

Compared to Samples A and C, identifying foreign objects in Sample B is challenging when examining the raw image in Fig 11 or the processed image with distortion compensation and ROI extraction in Fig 12. However, Fig 13, in which the influence of the exterior of the object is removed, accurately reveals the shapes of the plastic foreign objects within the sample. This result underscores the effectiveness of removing the exterior-related degradation to secure a sufficient dynamic range for capturing the shapes of foreign objects.

In contrast to Figs 9, 12, 15 demonstrates a less accurate ROI extraction. This is attributed to the diffraction patterns observed in the non-ROI region in the upper part of Fig 15. The increased exterior height of Sample C, compared with Samples A and B, exacerbates the diffraction and scattering effects along its boundaries. The ripple patterns visible outside the ROI in Fig 15 exemplify the following characteristics. Although the elevated exterior of Sample C produces a more distinct ROI region than Samples A and B, the proposed method shows limited improvement in ROI extraction for this scenario. These findings emphasize the necessity for a more precise ROI extraction technique to address such limitations effectively.

To quantitatively demonstrate the enhancement in the image quality achieved using the proposed preprocessing method, the image SNR was evaluated. For the original signal, the SNR was calculated using the region containing foreign bodies as the signal with the threshold level, based on the values from all pixels in the image. In contrast, the preprocessed images generated by the proposed method were reconstructed by isolating only the pixels within the ROI containing foreign bodies, resulting in an improved SNR. The preprocessing results for the three samples exhibited a significant SNR improvement: 5.0 dB for Sample A, 3.6 dB for Sample B, and 9.3 dB for Sample C. The preprocessed images were reconstructed by selecting only the pixels corresponding to foreign bodies within the ROI extraction region.

**Fig 17:**
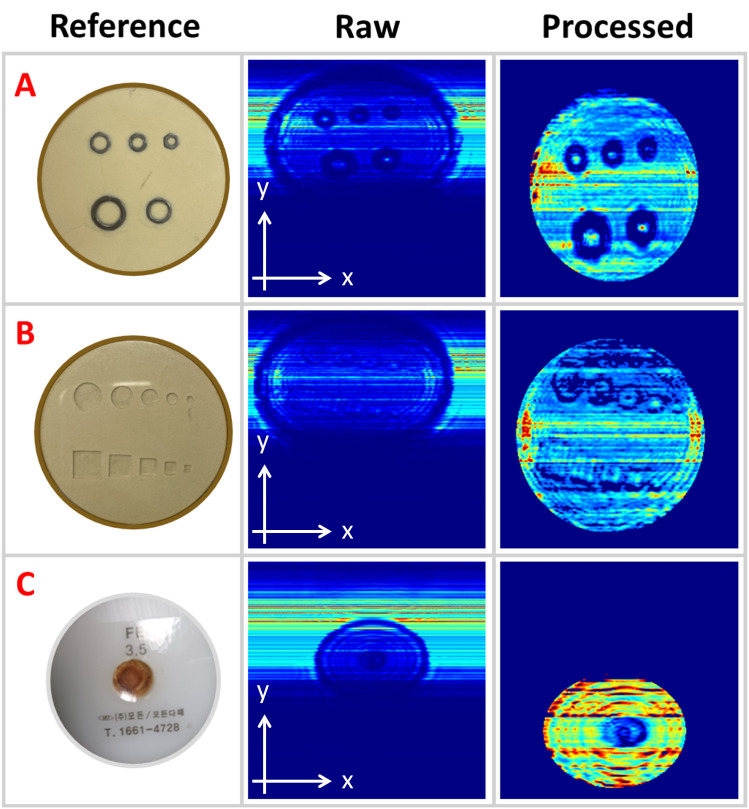
Processed images with FID algorithm for raw and preprocessed THz images for three samples.

Because the effectiveness of the proposed method depends on the sample image area and the corresponding acquired image region, it is challenging to apply metrics such as mean square error or peak signal-to-noise ratio, which rely on direct pixel-wise comparisons. The Fréchet inception distance (FID) is a metric used to evaluate the quality of images generated by generative models, such as generative adversarial networks [27]. It measures the distributional discrepancy between images preprocessed using the proposed method and actual sample images, providing an indication of their similarity. Inception-V3, a pretrained model for image recognition using Google, is utilized to extract feature vectors that capture information such as color patterns, shapes, and structural composition. The FID algorithm then calculates the mean vector and covariance matrix from the feature vectors for each image. By quantifying the distributional differences between two sets of images, the FID provides an assessment of image quality similar to human visual perception. Fig 17 presents the images subjected to the FID algorithm, demonstrating that the results of the proposed preprocessing method for all three samples exhibit greater similarity to the authentic sample images than to the original signal images. Table 1 provides a detailed comparison of these metrics between the raw and preprocessed images, highlighting the effectiveness of the method in extracting the ROI and improving the overall image clarity and fidelity.

**Table 1:** Image quality improvements achieved using the proposed preprocessing method.

Samples	A	B	C
**SNR Improvement**	5.0 dB	3.6 dB	9.3 dB
**FID (raw)**	474.197	443.128	493.719
**FID (preprocessed)**	396.106	386.352	390.498

## Conclusions

A signal preprocessing method is proposed for THz nondestructive transmission imaging systems aimed at the real-time detection of foreign objects on a conveyor belt. This method effectively removes the external appearance of a circular object by reconstructing only the interior of the sample. It incorporates a batch compensation process to account for the influences of the conveyor belt, object weight on the belt, and non-uniform beam profile. This approach overcomes the limitations of conventional conveyor belt compensation techniques and significantly improves the real-time image quality. This method effectively discards the external shape and isolates the ROI by exploiting rapid signal variations at the perimeter of the circular sample. In addition, the normalization process focuses solely on signals acquired from the interior of the object, resulting in a high image SNR and strong image correlation. The proposed method provides an effective preprocessing step for subsequent image processing using artificial intelligence, facilitating precise ROI extraction, compensating for system measurement effects, and producing images in the desired format, resolution, and size during reconstruction.

The proposed method serves as a preprocessing method for objects in the production stage, which are inspected individually as they move along a conveyor belt. As such, it is particularly well-suited for use in inspection processes during the final stages of production. However, implementing this method becomes challenging in scenarios in which multiple objects pass simultaneously or are stacked. This inherent limitation highlights the need for further research to extend the applicability of the proposed method to foreign-object detection in the earlier stages of mass production.
